# Modelling the cost-effectiveness of preventing major depression in general practice patients

**DOI:** 10.1017/S0033291713002067

**Published:** 2013-08-15

**Authors:** R. M. Hunter, I. Nazareth, S. Morris, M. King

**Affiliations:** 1 Department of Primary Care and Population Sciences, University College London Medical School, UK; 2 Department of Applied Health Research, University College London Medical School, UK; 3 Department of Mental Health Sciences, University College London Medical School, UK

**Keywords:** Cost-effectiveness, depression, economic evaluation, prevention, primary care

## Abstract

**Background:**

The prevention of depression is a key public health policy priority. PredictD is the first risk algorithm for the prediction of the onset of major depression. Our aim in this study was to model the cost-effectiveness of PredictD in depression prevention in general practice (GP).

**Method:**

A decision analytical model was developed to determine the cost-effectiveness of two approaches, each of which was compared to treatment as usual (TAU) over 12 months: (1) the PredictD risk algorithm plus a low-intensity depression prevention programme; and (2) a universal prevention programme in which there was no initial identification of those at risk. The model simulates the incidence of depression and disease progression over 12 months and calculates the net monetary benefit (NMB) from the National Health Service (NHS) perspective.

**Results:**

Providing patients with PredictD and a depression prevention programme prevented 15 (17%) cases of depression in a cohort of 1000 patients over 12 months and had the highest probability of being the optimal choice at a willingness to pay (WTP) of £20 000 for a quality-adjusted life year (QALY). Universal prevention was strongly dominated by PredictD plus a depression prevention programme in that universal prevention resulted in less QALYs than PredictD plus prevention for a greater cost.

**Conclusions:**

Using PredictD to identify primary-care patients at high risk of depression and providing them with a low-intensity prevention programme is potentially cost-effective at a WTP of £20 000 per QALY.

## Introduction

Reducing the prevalence of depression is a major public health challenge (Goldberg & Huxley, 1992). Depression has a significant impact on individuals and is associated with increased mortality and impaired social functioning (Cassano & Fava, 2002). It also results in significant costs to the health-care system and the economy. It was estimated that, in the year 2000, the annual National Health Service (NHS) cost of treating depression in England was £370 million (Thomas & Morris, 2003). The impact of depression on the lives of people who suffer from it, and on their families and friends, the NHS and the economy, means that prevention of depression is a key public health policy priority (HM Government, 2010).

Until now there has been no valid mechanism to identify people at risk of depression and provide them with prevention strategies. Previous cost-effectiveness analyses have focused predominantly on assisting patients who present with subthreshold depression, that is depressive symptoms that fall short of a depression diagnosis (Smit *et al*. 2006; Spek *et al*. 2008; Mihalopoulos *et al*. 2011), or screening to identify otherwise undiagnosed cases of depression and providing treatment (Valenstein *et al*. 2001; van den Berg *et al*. 2011). The use of low-intensity interventions to reduce the risk of major depression has generally been found to be both clinically and cost effective (Smit *et al*. 2006; Spek *et al*. 2008; Mihalopoulos *et al*. 2011; van den Berg *et al*. 2011). However, there has been no study of prevention for patients who are at risk of developing depression using a validated prediction algorithm.

Efforts at prevention have been hampered by lack of a reliable mechanism for identifying patients at risk of depression. PredictD is the first risk algorithm for the prediction of the onset of major depression. The algorithm was developed from 39 risk factors for depression in 5216 European general practice (GP) attendees who were not depressed at recruitment, including 1131 subjects from the UK (King *et al*. 2008). It was externally validated in 1732 patients in Chile who were not depressed at the time of recruitment. The risk algorithm contains nine factors (country, age, sex, educational level achieved, lifetime screen for depression, family history of psychological difficulties, physical health and mental health subscale scores on the 12-item Short Form Health Survey (SF-12; Jenkinson *et al*. 1997), unsupported difficulties in paid or unpaid work, and experiences of discrimination), which together provide an overall risk score. Those whose scores lie above the PredictD risk score threshold are considered to be at high risk of developing major depression in the next 12 months (King *et al*. 2008).

Decision analytical models use the best available information from a range of sources to estimate the costs and consequences of a particular intervention or policy, providing they take into account the uncertainty associated with the variables contained in the model (Akehurst *et al.* 2000). We modelled the cost-effectiveness of two approaches to depression prevention in GP attendees over 12 months compared to treatment as usual (TAU): (1) the PredictD risk algorithm plus a low-intensity prevention programme; (2) a universal prevention programme in which there was no initial identification of those at risk. Our principal difficulty was that there were no trial data available on the effectiveness of depression prevention programmes used in conjunction with a risk algorithm. Instead, we used the results of a meta-analysis of depression prevention programmes by Cuijpers *et al.* (2008) to calculate the probability that a risk prediction algorithm plus prevention programme for those at high risk is cost-effective compared to TAU over 12 months.

## Method

### Patient sample

Our model used a hypothetical baseline sample that we assumed had the same characteristics as a randomly selected sample of 1000 adults per treatment arm (over 18 years of age) attending GPs in the UK, with no current diagnosis of depression. The characteristics were considered to be the same as those of the 1131 patients (mean age 52 years; 66.3% female) in the original UK PredictD sample (King *et al.* 2008). The model assumes that patients are randomly allocated to one of the two intervention arms or the TAU arm so that 1000 patients who are representative of the adult primary-care population with no current diagnosis of depression enter each arm. The time horizon of the model is 12 months, a period of time in which the most reliable information on the progression of depression is available.

### Risk algorithm (PredictD) plus prevention programme

The 1000 patients are given the risk algorithm PredictD to complete, with the assistance of a primary-care nurse who calculates their PredictD risk score. Patients who score above the PredictD threshold for risk of depression are then directed towards a low-intensity depression prevention programme such as bibliotherapy, online cognitive behaviour therapy (CBT) or group therapy. Patients who score below the risk threshold on PredictD are not directed towards a prevention programme.

### Universal prevention

In universal prevention the whole population of interest, in this case GP attendees with no diagnosis of depression, are given access to or referred to an intervention without any screening. The strategy is most commonly used in the prevention of substance misuse (EMCDDA, 2012) but has also been proposed for the prevention of cardiovascular disease (Feenstra *et al.* 2011). It has the advantage that all patients at risk are directed towards the intervention, with minimal resources required for identification of patients, but the disadvantage that it is less targeted. All 1000 patients are directed towards a low-intensity prevention programme, such as online CBT or bibliotherapy, without any assessment of their risk of depression.

### TAU

Patients in the TAU group do not complete the risk algorithm or receive prevention. In all groups it is assumed that any patient who develops major depression will receive TAU from their GP, which might include prescription medication or referral to psychological therapies.

### Effectiveness of depression prevention programmes

There are no studies on the effectiveness of depression prevention programmes given to patients identified as being at high risk of developing depression by a risk algorithm. A meta-analysis by Cuijpers *et al.* (2008) of depression prevention programmes for people identified as having a high risk of major depression through other mechanisms, for example subthreshold depression or life events that increase the risk of depression, found that prevention programmes reduced the risk of developing depression by 22%. We calculated the odds ratio (OR) of developing depression from the meta-analysis (Cuijpers *et al.* 2008) and used the methodology set out in Briggs *et al*. (2006) separately for patients identified as at risk of depression and for universal programmes.

### Measuring outcomes

The outcome measure used in the model was quality-adjusted life years (QALYs). QALYs represent both the quality and quantity of health-related quality of life (HRQoL), quality being measured by utility scores. A utility score of 1 represents perfect health and a utility score of 0 death; negative values, representing states worse than death, are possible. QALYs are the recommended outcome for use in economic evaluations in the UK, as they are a common unit that allows for comparable decisions about resource allocation across different health conditions. In the UK, the National Institute for Health and Care Excellence (NICE) recommends that QALYs are calculated using utility scores generated by the Euroqol EQ-5D, a five-item, self-rated instrument covering mobility, self-care, usual activities, pain/discomfort and anxiety/depression (Kendrick *et al.* 2006; NICE, 2008).

Patients who were not depressed were assumed to have the utility of the general population of 0.86 (Kind *et al.* 1999). A search of the three databases of the Centre for Reviews and Dissemination (CRD, 2012) for studies with ‘depressed’ or ‘depression’ in the title and ‘EQ-5D’ in the text identified only two studies with UK EQ-5D utility scores for depression. A further two studies were identified from a systematic review (Peasgood *et al.* 2012). Utility scores for depressed and recovered patients were calculated from a weighted average from four UK trials (Peveler *et al.* 2005; Kendrick *et al.* 2006; Mann *et al.* 2009; Hollinghurst *et al.* 2010).

### Cost perspective

We modelled cost-effectiveness from the perspective of the NHS. Several sources were used to estimate NHS costs, using the 2010–2011 financial year as the base year. In instances where the standard error had not been reported, we assumed that it was equal to the mean, as recommended by Briggs *et al.* (2006).

### Cost of risk algorithm

The cost of a primary-care nurse completing the risk algorithm for 1000 patients was based on Personal Social Services Research Unit (PSSRU) costs for a GP nurse taking 15 min to administer the risk algorithm once to each patient. Training costs were calculated based on the estimate that it would cost £2000 to train enough primary-care nurses to complete the risk algorithm for 1000 patients.

### Cost of prevention programme

We assumed that the cost per patient of the prevention programme is likely to be between £0, representing freely available, online CBT (MoodGYM, 2010), and £200, similar to the highest cost per patient of implementing the computerized CBT package *Beating the Blues* (Kaltenthaler *et al.* 2002). Group therapy and bibliotherapy are likely to fall between these values, group therapy costing £140 per attendee if 10 sessions are attended (Curtis, 2012) and bibliotherapy potentially costing between £1 and £5 per patient.

### Cost of depression treatment

Treatment costs were calculated using published results to calculate the percentage of UK primary-care patients who receive prescription medication, psychological assistance or no treatment for depression (Weich *et al.* 2007). Hospital Episode Statistics (HES) for 2010–2011 combined with statistics on the prevalence of depression were used to estimate the percentage of patients who receive treatment from secondary care, that is in- and out-patient visits (HSCIC, 2011). Costs were calculated using the most recent NHS Reference Costs, PSSRU data, and data from the NHS Information Centre (DH PbR team, 2011; Curtis, 2012; HSCIC, 2012). A weighted average annual cost per patient to treat depression was calculated by multiplying the proportion of patients who access each type of treatment by the average annual cost of the treatment.

Treatment costs were calculated for patients in prevention and TAU arms for patients in the depressed state for the full 3, 6, 9 or 12 months that they were in the depressed state.

### Description of model

**Fig. 1.**
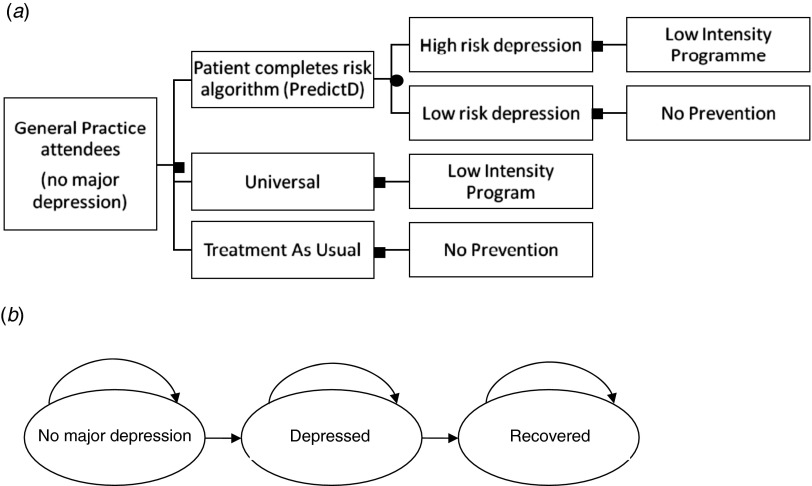
(*a*) Decision tree describing the three treatment arms and (*b*) Markov model of patients moving between no major depression, depressed and recovered states, as represented by the arrows.

The model used a decision tree (Fig. 1*a*) and a simple Markov model that simulates disease progression over 12 months (Fig. 1*b*). The Markov disease progression model is composed of three states: no major depression, depressed and recovered (Fig. 1*b*). Each model cycle represents 3 months and the model was run for 12 months (four cycles). All patients start in the ‘no major depression’ state and every 3 months either move to the next state or remain in the same state. Patients can spend 3, 6, 9 or 12 months in any of the three states. The rate of movement to each of the states, the transition probability, was determined in both models using previous reported rates of depression and recovery in a similar population over 12 months (Bottomley *et al.* 2010). The 6-month rates reported in Bottomley *et al.* (2010) were converted to 3-month rates and then translated into transition probabilities by calculating 1 minus the exponential of the 3-month probability.

For the PredictD plus prevention arm, the proportion of people identified as at risk of depression who would go on to develop depression if TAU only is provided and the OR of developing depression after receiving a prevention programme compared to TAU determine the proportion of patients who move from no depression into the depressed state.

In the universal prevention arm, the proportion of patients who go on to develop depression over 12 months is determined by the 1-year incidence of depression (Bottomley *et al.* 2010) and the OR for universal depression prevention programmes (Cuijpers *et al.* 2008).

The TAU group do not complete the risk algorithm, receive no preventative intervention and progress through to depression at an incidence rate that would be expected in the general primary-care population (King *et al.* 2008).

Because of the inevitability of false positives in the risk algorithm, a proportion of patients who receive prevention do so even though they would not have developed depression in the next 12 months. This is calculated based on the known specificity of PredictD (King *et al.* 2008).

As the time horizon for the model is 12 months, no discounting of costs or utilities has been included in the analysis. All yearly costs and utilities have been divided by four before being incorporated into the model so that they represent 3 months' worth of costs and QALYs.

The analysis was conducted using Microsoft Excel 2010.

### Cost-effectiveness analysis

We calculated the total costs and QALYs for the two prevention groups and TAU over 12 months from the NHS perspective. A probabilistic sensitivity analysis (PSA) was conducted to calculate the cost-effectiveness of each group using the net monetary benefit (NMB) approach, as described in Briggs *et al.* (2006). NMB is calculated as the total QALYs per arm, multiplied by a given willingness to pay (WTP) for a QALY, minus the total cost of the programme. NMBs were used to generate cost-effectiveness acceptability curves (CEACs). The CEACs are a summary of the proportion of times each option has the highest NMB for a given WTP for a QALY. All results are based on 10 000 simulations.

### Sensitivity analysis

The meta-analysis includes prevention studies from a range of patient groups and treatment locations. Given that PredictD was developed for GP attendees, we conducted an analysis comparing TAU with the combined OR for prevention programmes, where patients were recruited from GP patient lists only.

Cuijpers *et al.* (2008) observed some heterogeneity in their meta-analysis and therefore conducted some subgroup analyses. They found that interpersonal therapy (IP) was significantly more effective than other types of therapy. We conducted a PSA where the OR for IP is modelled separately.

To explore the relationship between the cost per patient of the prevention programme and its effectiveness at preventing depression, we calculated the maximum that a programme could cost for a given OR, if a decision maker is willing to pay £20 000 per QALY gained. This was calculated using the goal seek command in Excel 2010 and for 100 repetitions of each OR from 0 to 1 in increments of 0.01 using the PSA model.

Two additional PSAs were run to calculate the NMB and number of cases of depression prevented for the different PredictD risk score thresholds. The PredictD threshold of 0.133 (specificity 0.8, sensitivity 0.506) is the baseline value used in the model, as recommended by King *et al.* (2008). The higher two UK thresholds used in the two additional PSAs are 0.154 (specificity of 0.85, sensitivity of 0.458) and 0.183 (specificity of 0.9, sensitivity of 0.373).

The base case model was run with the assumption that all patients in the universal prevention arm access the prevention programme. To test what impact different assumptions about the percentage of patients accessing universal screening would have on the NMB, we ran 100 repetitions of the PSA model for each percentage point increase in patients accessing universal screening, from 0% to 100%. A further analysis was run that included a half cycle correction, so that half the patients in the depression state had the costs and disutility associated with depression for half of the cycle and the other half for the full cycle.

## Results

Table 1 summarizes the model inputs for the decision tree and the Markov disease progression model.

**Table 1.** Estimates used to determine parameters for the decision model

Parameter	Distribution for PSA^a^	Value (s.e.)	Source
Data for the decision tree			
Depression incidence UK (%/year)	Beta	8.8 (0.0084)	King *et al*. 2008
Sensitivity	Beta	0.506 (0.015)	King *et al*. 2008
Specificity	Beta	0.800 (0.012)	King *et al*. 2008
Odds ratio (OR)			
PredictD + prevention	Log-normal	0.66 (0.15)	Cuijpers *et al*. 2008
Universal prevention	Log-normal	0.9 (0.15)	Cuijpers *et al*. 2008
OR: sensitivity analysis			
Studies recruiting from GPs only	Log-normal	0.6 (0.35)	Cuijpers *et al*. 2008
PredictD + IP	Log-normal	0.14 (0.15)	Cuijpers *et al*. 2008
PredictD + prevention IP excluded	Log-normal	0.75 (0.13)	Cuijpers *et al*. 2008
Transition rates Markov model			
Depressed to recovered: months 0–6	Beta	0.18	Bottomley *et al*. 2010
Depressed to recovered: months 6–12	Beta	0.13	
Utility			
No major depression	Beta	0.86 (0.004)	Kind *et al*. 1999
Depression	Beta	0.58 (0.015)	Peveler *et al*. 2005; Kendrick *et al*. 2006; Mann *et al*. 2009; Hollinghurst *et al*. 2010
Recovered	Beta	0.79 (0.018)	Kendrick *et al*. 2006
Risk algorithm costs			
Cost of GP nurse (£/15 min)	Gamma	2.00	Curtis, 2012
Cost of training staff (£/patient)	Gamma	2.00	
Cost of prevention (£/patient)	Gamma	100	Kaltenthaler *et al*. 2002; Peveler *et al*. 2005
Average cost of depression treatment weighted average (£/patient/year)			
GP visits	Gamma	161.64	Dunn *et al*. 1999; Weich *et al*. 2007; Curtis, 2012
Prescription medication	Gamma	17.93	Weich *et al*. 2007; HSCIC, 2012
Psychological assistance/CBT	Gamma	137.09	Peveler *et al*. 2005; Weich *et al*. 2007; Curtis, 2012
In-patient stays	Gamma	21.60	DH PbR team, 2011; HSCIC, 2011
Out-patient visits	Gamma	2.09	DH PbR team, 2011; HSCIC, 2011
Total average cost		340.35	

PSA, Probabilistic sensitivity analysis; GP, general practice; IP, interpersonal therapy; CBT, cognitive behaviour therapy; s.e., standard error.

a Distributions based on Bayesian statistics. Beta distribution: constrained on the interval 0 to 1. Log-normal distribution: constrained on the interval 0 to positive infinity and is positively skewed (Briggs *et al*. 2006).

### Clinical and cost outcomes

Table 2 summarizes the costs and QALYs for the two prevention strategies and TAU, per 1000 patients over 1 year. Providing patients with PredictD and a depression prevention programme for those at high risk of depression prevented 15 cases of depression and resulted in two more QALYs than TAU for an additional cost to the NHS of £28 823. Universal prevention prevented eight cases of depression and resulted in one additional QALY compared to TAU, at an additional cost to the NHS of £85 356. Universal prevention was strongly dominated by PredictD and a prevention programme, in that universal prevention resulted in less QALYs at a higher cost.

**Table 2.** Clinical and cost outcomes from the model, where the yearly incidence of depression is 8.8% and the specificity and sensitivity of PredictD are 80% and 50.6% respectively

	Risk algorithm: low intensity	Universal	TAU
Clinical outcomes (per 1000 patients)			
Number given prevention programme	227	1000	–
Number depressed during 12 months	73	80	88
Number of cases of depression prevented	15	8	–
QALYs	849	847	846
Cost outcomes (£ per 1000 patients)			
Cost of completing risk algorithm	13 833	0	–
Total cost of prevention programme	19 684	87 507	–
Total cost of treatment for depression	22 763	25 307	27 458
Total NHS costs	56 281	112 814	27 458
NMB NHS costs (£ per patient)			
£20 000 per QALY	16 918	16 892	16 898

TAU, Treatment as usual QALY, quality-adjusted life year; NHS, National Health Service; NMB, net monetary benefit.

### NMB

**Fig. 2.**
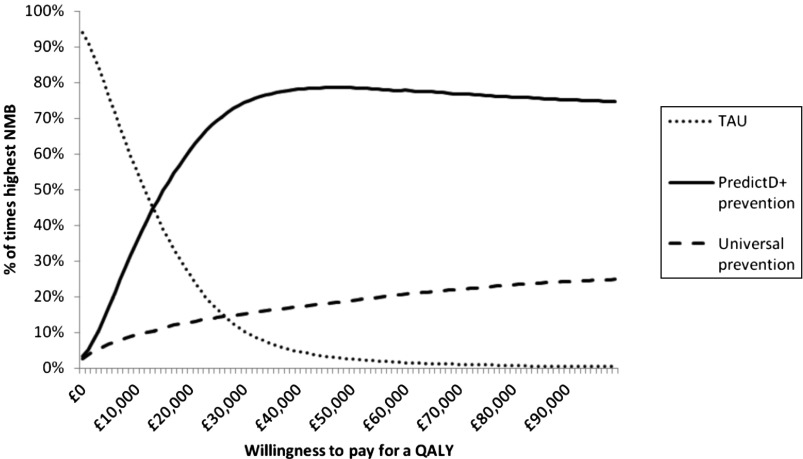
Percentage of cases where each option has the highest net monetary benefit (NMB) compared to treatment as usual (TAU): low-intensity interventions cost between £0 and £200 per patient (mean £100 and gamma distribution). QALY, quality-adjusted life year.

From an NHS perspective, PredictD and a prevention programme for patients at a high risk of depression has the highest NMB at a WTP of £20 000 per QALY (Table 2). Fig. 2 shows the probability that each option is the optimal choice for a given WTP for a QALY. At a WTP of £20 000 for a QALY, PredictD and a depression prevention programme has a 63% probability that it is the optimal choice compared to universal prevention and TAU.

### Studies recruiting GP patients

Two studies included in the meta-analysis recruited patients from GP lists only. Both studies identified patients at risk of depression and used a combination of group sessions, bibliotherapy, face-to-face therapy and telephone contact (Cuijpers *et al.* 2008). The combined OR of the two studies was 0.6 [95% confidence interval (CI) 0.3–1.2]. The total cost of giving non-depressed GP attendees PredictD, directing those at high risk towards a prevention programme and the cost of treatment for patients who develop depression was £56 332 to prevent 18 cases of depression. PredictD plus prevention has a 72% probability of being an optimal choice if a decision maker is willing to pay £20 000 for a QALY.

### IP

Studies where the prevention programme was IP had an OR of 0.13 (95% CI 0.04–0.42). If these studies are excluded when calculating the OR for prevention programmes, the new OR is 0.75 (95% CI 0.58–0.98). The total cost of giving non-depressed GP attendees PredictD, directing those at high risk towards IP and the cost of treatment for patients who develop depression was £49 714 to prevent 39 cases of depression, with an NMB of £16 995 at a WTP of £20 000 for a QALY.

If IP studies are excluded when calculating the OR, the total cost of giving non-depressed GP attendees PredictD, directing those at high risk towards a prevention programme and the cost of treatment for patients who develop depression was £58 435 to prevent 11 cases of depression, with an NMB of £16 902 at a WTP of £20 000 for a QALY.

### Cost of prevention programme versus effectiveness

**Fig. 3.**
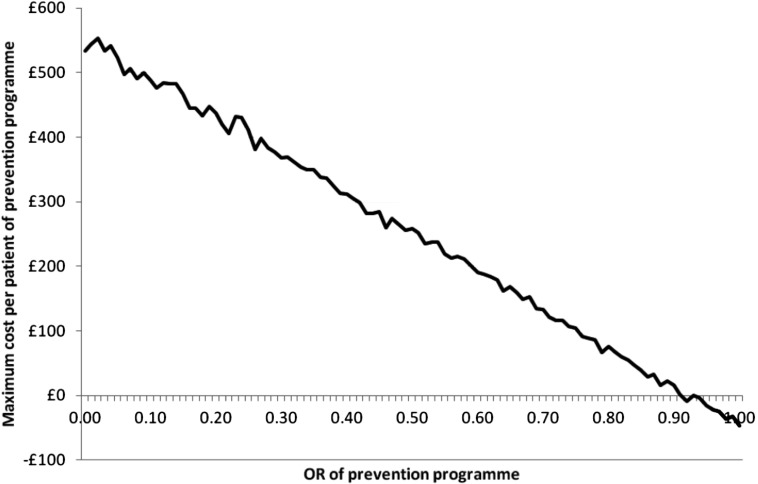
Maximum cost per patient for a prevention programme by odds ratio (OR). Willingness to pay (WTP) equals £20 000 per quality-adjusted life year (QALY) gained.

Fig. 3 summarizes the maximum that a prevention programme can cost for a given OR. Prevention programmes that prevent depression in 20% of people identified as at high risk (OR of 0.8) need to cost less than £75 per patient to be cost-effective at a threshold of £20 000 per QALY gained. Prevention programmes that prevent depression in less than 10% of patients identified as being at high risk are unlikely to be cost-effective as the required cost value is negative.

### Varying risk prediction algorithm thresholds

By providing a depression prevention programme to patients with a PredictD risk score above 0.154 (specificity of 0.85, sensitivity of 0.458), 14 cases of depression per 1000 patients are prevented over 12 months, with an NMB of £16 900. If the PredictD threshold 0.183 (specificity of 0.9, sensitivity of 0.373) is used, 11 cases of depression per 1000 patients are prevented over 12 months at an NMB of £16 904.

### Percentage of patients accessing universal prevention

At a WTP to pay for a QALY gained of £20 000 the PredictD risk algorithm plus a prevention programme for those identified as being at high risk remained the option that had the highest probability of being the optimal choice in all instances, unless less than 16% of patients offered universal screening accessed it. If 15% of patients access universal screening, there is a 47% probability that it is the optimal choice compared to 41% for the risk algorithm plus prevention and 12% for TAU.

### Half cycle correction

When a half cycle correction is included in the model, at a threshold of £20 000 per QALY gained, PredictD plus a prevention programme for those identified as being at high risk continues to have the highest NMB of £16 933. TAU has an NMB of £16 919 and a universal prevention programme an NMB of £16 856. The probability that PredictD plus a prevention programme is the optimal choice is 3 percentage points lower than the base-case scenario at 60%, at a WTP of £20 000 per QALY gained.

## Discussion

Identifying non-depressed GP attendees at risk of depression using the algorithm PredictD and providing them with access to a depression prevention programme is potentially more cost-effective than current practice at a WTP for a QALY of £20 000. It is also likely to be more cost-effective than providing all patients with a depression prevention programme with no identification of risk.

### Strengths and weaknesses

Although previous studies have examined the cost-effectiveness of identifying undiagnosed cases of depression or preventing depression in patients considered to be at high risk due to subthreshold depression or other clinical indicators, this is the first study to examine whether using a risk prediction algorithm, such as PredictD, to identify well people who are at high risk of depression, and then offering them a prevention programme, is cost-effective in averting the onset of depression.

The model contains a degree of uncertainty because it uses information from a variety of sources and is based on several assumptions. The effectiveness of prevention programmes was taken from a meta-analysis that pooled the effectiveness of different types of depression prevention programmes in different population groups that might not be representative of the GP population in the UK. Even when studies recruiting from GP surgeries only or significantly different studies were separated out, the conclusions remained the same.

Although the PredictD study recruited only 44% of the UK GP attenders asked to participate, compared to mostly higher responses in the other six participating European countries (King *et al.* 2008), the risk algorithm functioned with very similar accuracy across all counties. Thus, despite this lower response, it seems that the algorithm functions well and can be applied to all GP attenders in the UK, as modelled here.

In the model presented here, we have not specified what type of prevention programme is being implemented. Instead, we have summarized the maximum potential cost of a prevention programme for different levels of effectiveness, showing the wide range of values possible. We assumed a point estimate of the cost of the prevention programme of £100. Assuming an OR of 0.66, and as long as the prevention programme cost no more than £160 per patient receiving it, then the risk algorithm plus prevention programme was the most cost-effective option at a threshold of £20 000 per QALY gained, all else being equal. Additionally, in their meta-analysis, Cuijpers *et al.* (2008) did not find any significant difference between therapy types, except for IP. Although IP was shown to be significantly more effective, these results should be interpreted with caution, as they are based on only three studies of specific groups with short follow-up periods: two in postpartum women with 3 months of follow-up and one in adolescents with 6 months of follow-up. There were also only two studies in the meta-analysis that used universal prevention and neither of these was conducted in GP surgeries. As a result, it is not clear how universal prevention would function in GP surgeries, how feasible the implementation would be, or what would be the percentage of patients who would access the intervention. We were able to test the last point and found that, within reasonable limits, the percentage of patients accessing universal prevention has limited implications for the findings. Furthermore, the model does not make any estimates beyond 12 months. If the effects of different interventions were to persist beyond 12 months, then we may have underestimated the cost-effectiveness.

### Implications

Our analysis suggests that prevention of depression using a risk prediction algorithm such as PredictD in general practice and offering people at risk a prevention package is potentially cost-effective.

## References

[ref1] Akehurst R, Anderson P, Brazier J, Brennan A, Briggs A, Buxton M, Cairns J, Calvert N, Claxton K, Dixon S, Fryback D, Gallivan S, Green C, Lloyd A, McCabe C, Mitchell A, Nicholl J, O'Brien B, Roberts J, Sculpher M, Severens H, Sullivan S, Veenstra D; Signatories to the Consensus Statement (2000). *Decision Analytic Modelling in the Economic Evaluation of Health Technologies: A Consensus Statement*. Consensus Conference on Guidelines on Economic Modelling in Health Technology Assessment. PharmacoEconomics 17, 443–444 10977386 10.2165/00019053-200017050-00003

[ref2] Bottomley C, Nazareth I, Torres-Gonzalez F, Svab I, Maaroos HI, Geerlings MI, Xavier M, Saldivia S, King M (2010). Comparison of risk factors for the onset and maintenance of depression. Bristish Journal of Psychiatry 196, 13–17 10.1192/bjp.bp.109.067116 20044653

[ref3] Briggs A, Claxton K, Schulper M (2006). Decision Modelling for Health Economic Evaluation. Oxford University Press: Oxford

[ref4] Cassano P, Fava M (2002). Depression and public health: an overview. Journal of Psychosomatic Research 53, 849–857 12377293 10.1016/s0022-3999(02)00304-5

[ref5] CRD (2012). Centre for Reviews and Dissemination (www.crd.york.ac.uk/crdweb/). Accessed 28 March 2012

[ref6] Cuijpers P, van Straten A, Smit F, Mihalopoulos C, Beekman A (2008). Preventing the onset of depressive disorders: a meta-analytic review of psychological interventions. American Journal of Psychiatry 165, 1272–1280 18765483 10.1176/appi.ajp.2008.07091422

[ref7] Curtis L (2012). Unit Costs of Health and Social Care 2011. Personal Social Services Research Unit (PSSRU), University of Kent: Canterbury

[ref8] DH PbR team (2011). National Schedule of Reference Costs 2010–11. Department of Health Payment by Results team, Department of Health (https://www.gov.uk/government/publications/2010-11-reference-costs-publication). Accessed 10 May 2012

[ref9] Dunn RL, Donoghue JM, Ozminkowski RJ, Stephenson D, Hylan TR (1999). Longitudinal patterns of antidepressant prescribing in primary care in the UK: comparison with treatment guidelines. Journal of Psychopharmacology 13, 136–143 10475718 10.1177/026988119901300204

[ref10] EMCDDA (2012). Universal Prevention. European Monitoring Centre for Drugs and Drug Addiction (www.emcdda.europa.eu/html.cfm/index1578EN.html). Accessed 1 August 2012

[ref11] Feenstra TL, van Baal PM, Jacobs-van der Bruggen MO, Hoogenveen RT, Kommer GJ, Baan CA (2011). Targeted versus universal prevention. a resource allocation model to prioritize cardiovascular prevention. Cost Effectiveness and Resource Allocation 9, 14 21974836 10.1186/1478-7547-9-14 PMC3200148

[ref12] Goldberg DP, Huxley P (1992). Common Mental Disorders: A Biosocial Model. Routledge: London

[ref13] HM Government (2010). Healthy Lives, Healthy People: Our Strategy for Public Health in England. The Stationery Office: Norwich

[ref14] Hollinghurst S, Peters TJ, Kaur S, Wiles N, Lewis G, Kessler D (2010). Cost-effectiveness of therapist-delivered online cognitive-behavioural therapy for depression: randomised controlled trial. British Journal of Psychiatry 197, 297–304 20884953 10.1192/bjp.bp.109.073080

[ref15] HSCIC (2011). Hospital Episode Statistics, Admitted Patient Care - England, 2010–11: Primary Diagnosis. Health and Social Care Information Centre (www.hscic.gov.uk/pubs/hesadmitted1011). Accessed 10 May 2012

[ref16] HSCIC (2012). Prescription Cost Analysis: England 2011. Health and Social Care Information Centre (www.hscic.gov.uk/pubs/prescostanalysis2011). Accessed 10 May 2012

[ref17] Jenkinson C, Layte R, Jenkinson D, Lawrence K, Petersen S, Paice C, Stradling J (1997). A shorter form health survey: can the SF-12 replicate results from the SF-36 in longitudinal studies? Journal of Public Health Medicine 19, 179–186 9243433 10.1093/oxfordjournals.pubmed.a024606

[ref18] Kaltenthaler E, Shackley P, Stevens K, Beverley C, Parry G, Chilcott J (2002). A systematic review and economic evaluation of computerised cognitive behaviour therapy for depression and anxiety. Health Technology Assessment 6, 1–89 12433315 10.3310/hta6220

[ref19] Kendrick T, Peveler R, Longworth L, Baldwin D, Moore M, Chatwin J, Thornett A, Goddard J, Campbell M, Smith H, Buxton M, Thompson C (2006). Cost-effectiveness and cost-utility of tricyclic antidepressants, selective serotonin reuptake inhibitors and lofepramine: randomised controlled trial. British Journal of Psychiatry 188, 337–345 16582060 10.1192/bjp.188.4.337

[ref20] Kind P, Hardman G, Macran S (1999). UK Population Norms For EQ-5D. Centre for Health Economics Discussion Paper. University of York: York, UK

[ref21] King M, Walker C, Levy G, Bottomley C, Royston P, Weich S, Bellon-Saameno JA, Moreno B, Svab I, Rotar D, Rifel J, Maaroos HI, Aluoja A, Kalda R, Neeleman J, Geerlings MI, Xavier M, Carraca I, Goncalves-Pereira M, Vicente B, Saldivia S, Melipillan R, Torres-Gonzalez F, Nazareth I (2008). Development and validation of an international risk prediction algorithm for episodes of major depression in general practice attendees: the PredictD study. Archives of General Psychiatry 65, 1368–1376 19047523 10.1001/archpsyc.65.12.1368

[ref22] Mann R, Gilbody S, Richards D (2009). Putting the ‘Q’ in depression QALYs: a comparison of utility using EQ-5D and SF-6D health related quality of life measures. Social Psychiatry and Psychiatric Epidemiology 44, 134–136 10.1007/s00127-008-0463-5 19011721

[ref23] Mihalopoulos C, Vos T, Pirkis J, Smit F, Carter R (2011). Do indicated preventive interventions for depression represent good value for money? Australian and New Zealand Journal of Psychiatry 45, 36–44 21073354 10.3109/00048674.2010.501024

[ref24] MoodGYM (2010). The MoodGYM training programme: Mark III. Australian National University (www.moodgym.anu.edu.au/welcome). Accessed 12 November 2010

[ref25] NICE (2008). Guide to the Methods of Technology Appraisal *(reference N0515)*. National Institute for Health and Care Excellence: London, UK 27905712

[ref26] Peasgood T, Brazier J, Papaioannou D (2012). A Systematic Review of the Validity and Responsiveness of EQ-5D and SF-6D for Depression and Anxiety. Health Economics and Decision Science (HEDS) Discussion Paper, University of Sheffield

[ref27] Peveler R, Kendrick T, Buxton M, Longworth L, Baldwin D, Moore M, Chatwin J, Goddard J, Thornett A, Smith H, Campbell M, Thompson C (2005). A randomised controlled trial to compare the cost-effectiveness of tricyclic antidepressants, selective serotonin reuptake inhibitors and lofepramine. Health Technology Assessment 9, 1–134, iii. 15876362 10.3310/hta9160

[ref28] Smit F, Willemse G, Koopmanschap M, Onrust S, Cuijpers P, Beekman A (2006). Cost-effectiveness of preventing depression in primary care patients: randomised trial. British Journal of Psychiatry 188, 330–336 16582059 10.1192/bjp.188.4.330

[ref29] Spek V, Cuijpers P, Nyklicek I, Smits N, Riper H, Keyzer J, Pop V (2008). One-year follow-up results of a randomized controlled clinical trial on internet-based cognitive behavioural therapy for subthreshold depression in people over 50 years. Psychological Medicine 38, 635–639 18205965 10.1017/S0033291707002590

[ref30] Thomas CM, Morris S (2003). Cost of depression among adults in England in 2000. British Journal of Psychiatry 183, 514–519 14645022 10.1192/bjp.183.6.514

[ref31] Valenstein M, Vijan S, Zeber JE, Boehm K, Buttar A (2001). The cost-utility of screening for depression in primary care. Annals of Internal Medicine 134, 345–360 11242495 10.7326/0003-4819-134-5-200103060-00007

[ref32] van den Berg M, Smit F, Vos T, van Baal PH (2011). Cost-effectiveness of opportunistic screening and minimal contact psychotherapy to prevent depression in primary care patients. PLoS One 6, e22884 21853053 10.1371/journal.pone.0022884 PMC3154255

[ref33] Weich S, Nazareth I, Morgan L, King M (2007). Treatment of depression in primary care. Socio-economic status, clinical need and receipt of treatment. British Journal of Psychiatry 191, 164–169 17666502 10.1192/bjp.bp.106.032219

